# Asymptotic Thermodynamics for Chemical Reaction Networks with Fast-Slow Kinetics

**DOI:** 10.3390/e28070825

**Published:** 2026-07-20

**Authors:** Liangrong Peng, Liu Hong

**Affiliations:** 1 School of Computer and Big Data, Minjiang University, Fuzhou 350108, China; 2 School of Mathematics, Sun Yat-sen University, Guangzhou 510275, China

**Keywords:** chemical reaction, non-equilibrium thermodynamics, asymptotic expansion, time scale separation, Michaelis–Menten reaction

## Abstract

We present a systematic derivation of asymptotic expansions of nonequilibrium thermodynamics for chemical reaction networks (CRNs) based on singular perturbation theory. For a general reversible CRN with fast–slow kinetics, we obtain the first and second laws of thermodynamics for the asymptotic expansion model. We derive composite expansions of the enthalpy, entropy, entropy production rate, and relative entropy. The slow-varying outer parts of these thermodynamic quantities capture the long-time trend, while the fast-varying corrected inner parts decay to zero as the fast time variable tends to infinity. The convergence order of these quantities is determined by the local Lipschitz properties of the respective functions. The enthalpy retains the same convergence order as the kinetic variables, whereas the entropy, relative entropy, and entropy production rate involve logarithmic terms that cause their gradients to diverge when some concentrations approach zero, reducing their theoretical convergence order by one. The general theory is validated on the reversible Michaelis–Menten reaction, for which both leading-order and first-order matched asymptotic expansions are obtained analytically. Numerical simulations confirm the uniform accuracy of the composite thermodynamic approximations and further reveal that the entropy production rate converges with a higher order than theoretically predicted. The results demonstrate that the composite expansion provides a rigorous and physically consistent tool for analyzing energy and entropy balances in multiscale CRNs.

## 1. Introduction

Chemical reaction networks (CRNs) govern a range of processes in chemical engineering, material science, biology, etc. [1]. In many of these systems, the underlying kinetics span widely separated time scales, a few fast reactions approach equilibrium or quasi-steady states on a scale orders of magnitude shorter than the slow reactions. Singular perturbation theory provides analytical tools for describing CRNs that evolve on two or more well-separated time scales [2,3,4]. By identifying a proper small parameter, typically the ratio of the slowest to the fastest reaction rates, one can construct matched asymptotic expansions that decompose the full dynamics into a simplified long-time part and its fast corrections. This approach not only yields quantitatively accurate approximations, but also reveals the multiscale structure inherent in the kinetics.

Singular perturbation methods, including the quasi-steady-state approximation (QSSA) and partial equilibrium approximation (PEA), have been extensively applied to model reduction in chemical kinetics. QSSA (also known as pseudo-steady-state hypothesis, PSSH [2,5]) operates on species concentrations, whereas PEA (also known as quasiequilibrium hypothesis [6]) operates on reaction steps. Both approximations can be systematically derived and justified through matched asymptotic expansions [5,7]. For example, QSSA was analyzed as a case study in singular perturbation by Segel and Slemrod [2], who demonstrated that proper nondimensionalization reveals a small parameter different from the one conventionally assumed. Heineken et al. [8] illustrated the accuracy of parameter estimation through enzymatic kinetics reduced by QSSA, providing quantitative guidance on optimal experimental design. Bowen et al. [9] refined QSSA with the aid of singular perturbation methods. Gorban and Shahzad [6] later showed that the combination of QSSA and quasiequilibrium hypothesis leads to the generalized mass-action law based on the Michaelis–Menten–Stueckelberg theorem. Yong [3] proposed a framework to justify PEA based on the conservation–dissipation formalism, while Huang et al. [4] further applied PEA to signal transduction cascades. Feliu et al. [10] characterized Tikhonov–Fenichel parameter values for CRNs, where reduction occurs by turning off reactions or removing species. More broadly, the theory of slow invariant manifolds [11,12] and computational singular perturbation [13] offer effective methods to identify attracting low-dimensional manifolds on which long-time dynamics unfold.

In parallel, nonequilibrium thermodynamics provides a universal language for characterizing energy and entropy flows in CRNs [14,15]. The balance of enthalpy at constant temperature and pressure describes the first law of thermodynamics, whereas the entropy, the entropy production rate [16,17], and the relative entropy [18,19] quantify the second law of thermodynamics. Shear’s theorem [20,21] established that relative entropy serves as a Lyapunov function for mass-action chemical kinetics, revealing that closed CRNs will always approach to the equilibrium steady state. Although kinetic reduction methods have been extensively developed, their thermodynamic implications have received less attention. Recent work has begun to bridge the reduced kinetics and thermodynamic perspectives. Öttinger [22] developed a model-reduction procedure that preserves the underlying thermodynamic structure by constructing the Poisson and irreversible brackets of nonequilibrium thermodynamics on an invariant manifold. Grmela [23] examined the roles of energy and entropy in multiscale reduction, showing that entropy acts as both a repository for unresolved energy and a filter revealing emergent features of the reduced description. Busiello et al. [24] proposed a general framework for coarse-graining the entropy production in systems with multiple coupled processes and separated time scales, highlighting the finite correction that arises when fast processes do not fully equilibrate. Ge et al. [25] established a martingale structure for general thermodynamic functionals of diffusion processes under second-order averaging, providing a rigorous stochastic foundation for the decomposition of entropy production into regular and anomalous contributions. Peng and Hong [26] elucidated quantitative connections between the thermodynamics of the full mass-action kinetics and those of models reduced by PEA and QSSA, showing that the free-energy dissipation of the reduced models is not preserved to be non-negative. Zhang et al. [27] generalized the above results from closed CRNs to open CRNs. These findings highlight the need for a thermodynamic perspective when evaluating the accuracy of reduced kinetic models.

Despite the progress achieved on the separate fronts of singular perturbation methods and thermodynamics of CRNs, a systematic investigation of the asymptotic expansions of thermodynamic quantities has, to the best of our knowledge, not been undertaken. In this work, we derive asymptotic expansions of nonequilibrium thermodynamics for general reversible CRNs with fast–slow kinetics. In Section 2, after formulating multiscale mass-action equations and introducing the method of matched asymptotic expansions and nonequilibrium thermodynamics of CRNs, we derive the asymptotic expansions of nonequilibrium quantities. Section 3 applies the framework to the reversible Michaelis–Menten reaction, for which the leading-order and first-order matched asymptotic expansions are obtained. The numerical results confirm the theoretical predictions. Section 4 concludes with a brief discussion.

## 2. Asymptotic Expansions of Nonequilibrium Thermodynamics of CRNs

In this section, we first introduce the multiscale chemical mass-action kinetics and its singular perturbation methods. Then we present the thermodynamics for the full CRNs and investigate its asymptotic expansions.

### 2.1. Multiscale Chemical Mass-Action Equations

We consider a general CRN composed of *N* species $S=(S_{1},S_{2},\cdots ,S_{N})$, which is undergoing *M* reversible reactions

(1) $$S\nu^{+}\underset{k^{-}}{\overset{k^{+}}{⇌}}S\nu^{-},$$

where $\nu^{\pm }={\left[\left(\nu_{ij}^{\pm }\right)\right]}_{N\times M}\geq 0$ are stoichiometric matrix, $k^{\pm }=\left(k_{1}^{\pm },k_{2}^{\pm },\cdots ,k_{M}^{\pm }\right)\geq 0$ are reaction rate vector. The concentration vector of species is denoted as $c\left(t\right)={\left(c_{1},c_{2},\cdots ,c_{N}\right)}^{T}$, whose evolution is governed by the following ordinary differential equations (ODEs) [17]

(2) $$\frac{dc}{dt}=\nu R\left(c\right).$$

Here the vector $R\left(c\right)={\left(R_{1}\left(c\right),R_{2}\left(c\right),\cdots ,R_{M}\left(c\right)\right)}^{T}$ is the net reaction rate function, which is the difference between the forward and backward reaction rate functions, $R\left(c\right)=R^{+}\left(c\right)-R^{-}\left(c\right)$, and the matrix $\nu =\nu^{-}-\nu^{+}$. In this work, we assume that the reactions in (1) are elementary with constant temperature and constant pressure. Based on the mass-action law, the forward and backward reaction rate functions have a polynomial form of concentrations

(3) $$R_{j}^{+}\left(c\right)=k_{j}^{+}\prod_{l=1}^{N}c_{l}^{\nu_{lj}^{+}},\;R_{j}^{-}\left(c\right)=k_{j}^{-}\prod_{l=1}^{N}c_{l}^{\nu_{lj}^{-}}.$$

The CRN (1) obeys detailed balance if and only if there exists a positive equilibrium state $c_{e}>0$ such that

(4) $$R^{+}\left(c_{e}\right)=R^{-}\left(c_{e}\right),$$

i.e., the forward and backward reaction rates coincide.

We focus on the case where Equation (2) can be nondimensionalized to exhibit two well separated time scales—fast and slow ones:

(5) $$\begin{array}{cc} \; & \frac{dx}{dt}=f\left(x,y\right),\;x\in R^{m},\\ \; & \epsilon \frac{dy}{dt}=g\left(x,y\right),\;y\in R^{n},\\ \; & x(0;\epsilon )=a,\;y(0;\epsilon )=b. \end{array}$$

Here 0<ϵ≪1 is a small parameter; both the slow variable x and the fast variable y are dimensionless concentrations of chemical reactants; $f:R^{m}\times R^{n}\to R^{m}$ and $g:R^{m}\times R^{n}\to R^{n}$ are dimensionless net reaction rates. The initial values a and b are assumed to be non-negative constant vectors, independent of ϵ. The solution to Equation (5) will be denoted by $\left(x^{\epsilon }\left(t\right),\;y^{\epsilon }\left(t\right)\right)$.

### 2.2. Singular Perturbation Methods for Multiscale ODEs

Constructing asymptotic expansions for the solution $\left(x^{\epsilon }\left(t\right),\;y^{\epsilon }\left(t\right)\right)$ not only provides an accurate approximation but, more importantly, offers a powerful analytical framework to reveal the multiscale structure inherent in the system. The presence of the small parameter ϵ in the highest-order term of Equation (5) renders the problem into a singular perturbation one [28].

Outer solution. Setting ϵ=0 in (5) reduces the full ODEs to the differential-algebraic system

(6a) $$\begin{array}{cc} \; & g({\bar{x}}_{0},{\bar{y}}_{0})=0, \end{array}$$

(6b) $$\begin{array}{cc} \; & \frac{d{\bar{x}}_{0}}{dt}=f\left({\bar{x}}_{0},{\bar{y}}_{0}\right),\;{\bar{x}}_{0}\left(0\right)=a. \end{array}$$

Assume that there exists a function $\phi :R^{m}\to R^{n}$, $\phi \left({\bar{x}}_{0}\right)={\bar{y}}_{0}$, such that $g({\bar{x}}_{0},\phi \left({\bar{x}}_{0}\right))=0$ is satisfied for each fixed ${\bar{x}}_{0}$. Then the ODE in (6b) is decoupled from (6a) after substituting the relation ${\bar{y}}_{0}=\phi \left({\bar{x}}_{0}\right)$. Solving it provides the leading-order outer solution $\left({\bar{x}}_{0}\left(t\right),\;{\bar{y}}_{0}\left(t\right)=\phi \left({\bar{x}}_{0}\left(t\right)\right)\right)$. However, this outer solution generally fails to satisfy the initial condition for y, indicating an initial layer adjacent to t=0.

Inner solution. Inside the initial layer, we introduce the scaled time variable τ=t/ϵ. Denote the rescaled solution to Equation (5) as $\left(x^{\epsilon }\left(\epsilon \tau \right),\;y^{\epsilon }\left(\epsilon \tau \right)\right)=\left({\hat{x}}^{\epsilon }\left(\tau \right),\;{\hat{y}}^{\epsilon }\left(\tau \right)\right)$. Expanding ${\hat{x}}^{\epsilon }\left(\tau \right)={\hat{x}}_{0}\left(\tau \right)+\epsilon {\hat{x}}_{1}\left(\tau \right)+\cdots$ and ${\hat{y}}^{\epsilon }\left(\tau \right)={\hat{y}}_{0}\left(\tau \right)+\epsilon {\hat{y}}_{1}\left(\tau \right)+\cdots$, inserting it into Equation (5) and then letting ϵ=0, we arrive at

(7) $$\begin{array}{cc} \; & \frac{d{\hat{x}}_{0}}{d\tau }=0,\;{\hat{x}}_{0}\left(0\right)=a,\\ \; & \frac{d{\hat{y}}_{0}}{d\tau }=g\left({\hat{x}}_{0},{\hat{y}}_{0}\right),\;{\hat{y}}_{0}\left(0\right)=b, \end{array}$$

whose solution $\left({\hat{x}}_{0}\left(\tau \right)\equiv a,\;{\hat{y}}_{0}\left(\tau \right)\right)$ is called the leading-order inner solution.

Solution Matching. The inner limit of the outer solution and the outer limit of the inner solution should be compatible:

(8) $$\lim_{t\to 0}\left({\bar{x}}_{0}\left(t\right),\;{\bar{y}}_{0}\left(t\right)\right)=\lim_{\tau \to \infty }\left({\hat{x}}_{0}\left(\tau \right),\;{\hat{y}}_{0}\left(\tau \right)\right),$$

which gives ${\bar{y}}_{0}\left(0\right)=\phi \left(a\right)={\hat{y}}_{0}\left(\infty \right)\equiv \lim_{\tau \to \infty }{\hat{y}}_{0}\left(\tau \right)$. Then, we have the corrected inner solutions ${\tilde{x}}_{0}\left(\tau \right)\equiv {\hat{x}}_{0}\left(\tau \right)-{\bar{x}}_{0}\left(0\right)=0$ and ${\tilde{y}}_{0}\left(\tau \right)\equiv {\hat{y}}_{0}\left(\tau \right)-{\bar{y}}_{0}\left(0\right)$. Adding them to the outer solution yields the leading-order composite solution $\left({\bar{x}}_{0}\left(t\right)+{\tilde{x}}_{0}\left(\tau \right),\;{\bar{y}}_{0}\left(t\right)+{\tilde{y}}_{0}\left(\tau \right)\right)$.

Higher-order asymptotic expansions [28] can be deduced in a similar manner. Please refer to Section 3.1 for a detailed example. The matched solution is uniformly valid throughout the time domain [0,T] [28], which is elegantly decomposed as

(9) $$\begin{array}{cc} \; & x^{\epsilon }\left(t\right)={\bar{x}}^{\epsilon }\left(t\right)+{\tilde{x}}^{\epsilon }\left(\tau \right)+O\left(\epsilon^{\alpha }\right),\\ \; & y^{\epsilon }\left(t\right)={\bar{y}}^{\epsilon }\left(t\right)+{\tilde{y}}^{\epsilon }\left(\tau \right)+O\left(\epsilon^{\alpha }\right), \end{array}$$

where α≥1 is the order, ${\bar{x}}^{\epsilon }\left(t\right)$ and ${\bar{y}}^{\epsilon }\left(t\right)$ are the outer solutions, ${\tilde{x}}^{\epsilon }\left(\tau \right)$ and ${\tilde{y}}^{\epsilon }\left(\tau \right)$ are the corrected inner solutions, respectively. In what follows, we will omit the dependence of $\left({\bar{x}}^{\epsilon }\left(t\right),\;{\bar{y}}^{\epsilon }\left(t\right),\;{\tilde{x}}^{\epsilon }\left(\tau \right),\;{\tilde{y}}^{\epsilon }\left(\tau \right)\right)$ on ϵ.

We will illustrate the idea of composite solutions in detail by the Michaelis–Menten reaction in Section 3.

### 2.3. Thermodynamics for CRNs

Here the nonequilibrium thermodynamics for CRNs is introduced in a nutshell by the first and second laws.

The first law of thermodynamics. For CRNs (1) at constant pressure, the first law of thermodynamics [15,17] degenerates into the fact that the internal energy density *U* equals the enthalpy density *H* minus the pressure P, U(t)=H(t)−P. Hereinafter, the word density is omitted from the expression for simplicity.

Enthalpy. The enthalpy of CRNs (1) is a linear function of concentrations:

(10) $$H\left(t\right)=c\cdot h^{\circ },$$

where $h^{\circ }$ is the standard-state enthalpies of formation, and any constant reference enthalpy is set to zero for simplicity. The enthalpy evolution follows directly from the kinetics (2):

(11) $$\frac{dH}{dt}=h^{\circ }\cdot \nu R\left(c\right).$$

The second law of thermodynamics.

Entropy. In contrast to enthalpy, the entropy of CRNs (1) is a nonlinear function composed of the mixing entropy and the formation entropy [15,17]:

(12) $$\mathrm{Ent}\left(t\right)=\underset{\mathrm{mixing}\;\mathrm{entropy}}{\underset{︸}{-Rc\cdot (\ln c-1)}}+\underset{\mathrm{formation}\;\mathrm{entropy}}{\underset{︸}{c\cdot s^{\circ }}},$$

where R is the gas constant, 1 is a vector of ones, $s^{\circ }$ is the standard-state entropy of formation.

Entropy evolution. The change rate of entropy is decomposed into two terms:

(13) $$\frac{d}{dt}\mathrm{Ent}\left(t\right)=\underset{J_{f}\left(t\right)}{\underset{︸}{s^{\circ }\cdot \nu R(c)-RR\left(c\right)\cdot \ln\frac{\kappa^{+}}{\kappa^{-}}}}+\underset{\mathrm{epr}(t)\geq 0}{\underset{︸}{R\left[R^{+}\left(c\right)-R^{-}\left(c\right)\right]\cdot \ln\frac{R^{+}\left(c\right)}{R^{-}\left(c\right)}}},$$

where $J_{f}\left(t\right)$ is the entropy flow rate, and the nonnegative epr(t) is the entropy production rate, whose positivity for irreversible processes is the direct statement of the second law.

Relative entropy. The relative entropy is the Kullback–Leibler (KL) divergence between the time-dependent state c(t) and the steady state $c_{e}$:

(14) $$F\left(t\right)=\mathrm{RT}D_{\mathrm{KL}}\left(c∥c_{e}\right)=\mathrm{RT}\left(c\cdot \ln\frac{c}{c_{e}}-c\cdot 1+c_{e}\cdot 1\right)\geq 0,$$

where the non-negativity of F(t) is guaranteed by the Gibbs inequality, and F(t)=0 if and only if $c\left(t\right)=c_{e}$. The free energy dissipation rate is introduced as $f_{d}\left(t\right)=-dF/dt$. That is,

(15) $$f_{d}\left(t\right)=\mathrm{RT}R\left(c\right)\cdot \ln\left(\frac{R^{+}\left(c\right)R^{-}\left(c_{e}\right)}{R^{-}\left(c\right)R^{+}\left(c_{e}\right)}\right)\geq 0.$$

Since dF/dt≤0, the relative entropy F(t) decreases monotonically along any solution of Equation (2), and attains its global minimum only when $c\left(t\right)=c_{e}$. Thus, F(t) acts as a Lyapunov function for reversible CRNs (1) under the condition of detailed balance, which is known as the Shear’s theorem [20,21].

Given the local detailed balance condition, the relative entropy F(t) is equal to the Gibbs free energy G(t) up to a constant. We therefore prefer to discuss the relative entropy rather than the Gibbs free energy in the subsequent sections.

### 2.4. Asymptotic Expansions of Thermodynamics for CRNs

Denote the solution of Equation (2) as $c^{\epsilon }\left(t\right)$, where ϵ is a small parameter. To avoid lengthy mathematical notation, we assume that the concentration vector admits the composite representation at order α(α≥1)

(16) $$c^{\epsilon }\left(t\right)=\bar{c}\left(t\right)+\tilde{c}\left(\tau \right)+O\left(\epsilon^{\alpha }\right),$$

where τ=t/ϵ, $\bar{c}\left(t\right)$ is the outer solution and $\tilde{c}\left(\tau \right)$ is the corrected inner solution satisfying $\tilde{c}\left(\tau \right)\to 0$ as τ→∞. Since the steady state is dependent on ϵ, we denote the equilibrium of Equation (2) as $c_{e}^{\epsilon }$. At the same time, we denote the steady state of the outer solution by ${\bar{c}}_{e}$, which typically differs from the equilibrium $c_{e}^{\epsilon }$ of the original system by an $O(\epsilon^{\alpha })$ term.

For any thermodynamic function $F\left[\cdot \right]\in C^{1}\left({\left(0,\infty \right)}^{N}\right)$, we denote the exact value as $F^{\epsilon }\left(t\right)\equiv F\left[c^{\epsilon }\left(t\right)\right]$. We substitute the composite expansion (16) into F to obtain its approximation

(17) $$F^{\epsilon }\left(t\right)=F\left[\bar{c}\left(t\right)+\tilde{c}\left(\tau \right)\right]+O\left(\epsilon^{\beta }\right),$$

where the convergence order β is dictated by the local Lipschitz properties of the thermodynamic function F[·] near the concentration trajectory. For $F\in C^{1}$, the Mean Value Theorem yields

$$\left|F[c+\delta c]-F[c]\right|\leq \max_{\xi \in [c,c+\delta c]}∥\nabla F\left(\xi \right)∥\;∥\delta c∥.$$

Hence, if ∇F remains bounded along the trajectory and its composite approximation, the error of F inherits the order of the concentration expansion, i.e., β=α. This is the case for the enthalpy *H*, which is linear in c and thus satisfies the Lipchitz condition.

However, the entropy Ent, relative entropy *F*, and entropy production rate epr behave differently. In singularly perturbed problems, some species may transiently approach zero within the initial layer, such as the product [P] in the MM reaction. In these regions, the gradients of Ent, *F* and epr involve logarithmic terms $\ln c_{i}$, which diverge as $c_{i}\to 0$. Consequently, the local Lipschitz constant becomes as large as |lnϵ|. Even if the concentration approximation satisfies $∥c^{\epsilon }-\left(\bar{c}+\tilde{c}\right)∥=O\left(\epsilon^{\alpha }\right)$, the error of Ent, *F* and epr can be amplified to $O(\epsilon^{\alpha }|\ln\epsilon |)$. Therefore, the theoretical convergence order of Ent, *F* and epr is one order lower than that of concentrations, i.e., β=α−1.

Now we proceed to derive the asymptotic expansions of thermodynamics for CRNs (1). The thermodynamic function $F^{\epsilon }\left(t\right)$ enjoys the following composite decomposition:

(18) $$F^{\epsilon }\left(t\right)=\bar{F}\left(t\right)+\tilde{F}\left(\tau \right)+O\left(\epsilon^{\beta }\right),$$

where the outer part is defined as $\bar{F}\left(t\right)\equiv F\left[\bar{c}\left(t\right)\right]$, and the inner correction is $\tilde{F}\left(\tau \right)\equiv F\left[\bar{c}\left(t\right)+\tilde{c}\left(\tau \right)\right]-F\left[\bar{c}\left(t\right)\right]$. This construction guarantees that the slow-varying outer part $\bar{F}\left(t\right)$ captures the long-time trend, while the fast-varying corrected inner part $\tilde{F}\left(\tau \right)\to 0$ as τ→∞ due to the continuity of F[·].

**Remark** **1.** *The above expressions are exact to $O(\epsilon^{\beta })$ and do not rely on linearizing F[·] around $\bar{c}$. They automatically incorporate higher-order terms in $\tilde{c}$, which are essential to recover the correct thermodynamic behavior inside the initial layer.*

Enthalpy. Thanks to the linear dependence of the enthalpy (10) on the concentration vector, we can derive its asymptotic expansion as

(19) $$H^{\epsilon }\left(t\right)=\bar{H}\left(t\right)+\tilde{H}\left(\tau \right)+O\left(\epsilon^{\alpha }\right),\;\bar{H}\left(t\right)=\bar{c}\left(t\right)\cdot h^{\circ },\;\tilde{H}\left(\tau \right)=\tilde{c}\left(\tau \right)\cdot h^{\circ },$$

where $\bar{H}\left(t\right)$ and $\tilde{H}\left(\tau \right)$ are the outer and corrected inner contributions of the enthalpy, called as the outer enthalpy and corrected inner enthalpy, respectively.

Entropy. For the entropy function Ent[c] in Equation (12), we obtain:

(20) $$\begin{array}{cc} \; & {\mathrm{Ent}}^{\epsilon }\left(t\right)=\bar{\mathrm{Ent}}\left(t\right)+\tilde{\mathrm{Ent}}\left(\tau \right)+O\left(\epsilon^{\beta }\right), \end{array}$$

(21) $$\begin{array}{cc} \; & \bar{\mathrm{Ent}}\left(t\right)=-R\bar{c}\left(t\right)\cdot \left[\ln\bar{c}\left(t\right)-1\right]+\bar{c}\left(t\right)\cdot s^{\circ }, \end{array}$$

where $\bar{\mathrm{Ent}}\left(t\right)$ is the outer entropy, and $\tilde{\mathrm{Ent}}\left(\tau \right)=-R\left[\left(\bar{c}\left(t\right)+\tilde{c}\left(\tau \right)\right)\cdot \ln\left(\bar{c}\left(t\right)+\tilde{c}\left(\tau \right)\right)-\bar{c}\left(t\right)\ln\bar{c}\left(t\right)-\tilde{c}\left(\tau \right)\right]+\tilde{c}\left(\tau \right)\cdot s^{\circ }$ is the inner correction.

Entropy production rate. Analogously, for the entropy production rate epr[c] given in Equation (13),

(22) $$\begin{array}{cc} \; & {\mathrm{epr}}^{\epsilon }\left(t\right)=\bar{\mathrm{epr}}\left(t\right)+\tilde{\mathrm{epr}}\left(\tau \right)+O\left(\epsilon^{\beta }\right), \end{array}$$

(23) $$\begin{array}{cc} \; & \bar{\mathrm{epr}}\left(t\right)=R\left[R^{+}\left(\bar{c}\left(t\right)\right)-R^{-}\left(\bar{c}\left(t\right)\right)\right]\cdot \ln\frac{R^{+}\left(\bar{c}\left(t\right)\right)}{R^{-}\left(\bar{c}\left(t\right)\right)}\geq 0, \end{array}$$

where the outer entropy production rate $\bar{\mathrm{epr}}\left(t\right)$ is nonnegative, and the inner correction is $\tilde{\mathrm{epr}}\left(\tau \right)=\mathrm{epr}\left[\bar{c}\left(t\right)+\tilde{c}\left(\tau \right)\right]-\mathrm{epr}\left[\bar{c}\left(t\right)\right]$. The entropy flow rate $J_{f}\left(t\right)$ then follows from the entropy balance $J_{f}\left(t\right)=\frac{d}{dt}\mathrm{Ent}\left(t\right)-\mathrm{epr}\left(t\right)$.

The asymptotic expansion of the relative entropy is distinct from the other thermodynamic functions discussed above. In the following, the reference state of relative entropy is carefully analyzed.

Relative entropy. Recall that the relative entropy is the KL divergence between $c^{\epsilon }\left(t\right)$ and $c_{e}^{\epsilon }$, $F^{\epsilon }\left(t\right)=\mathrm{RT}D_{\mathrm{KL}}\left(c^{\epsilon }\left(t\right)∥c_{e}^{\epsilon }\right)$. When focused on the outer solution, it is natural to introduce the outer part $\bar{F}\left(t\right)$ of the relative entropy by the KL divergence between $\bar{c}\left(t\right)$ and its steady state ${\bar{c}}_{e}$:

(24) $$\bar{F}\left(t\right)=\mathrm{RT}D_{\mathrm{KL}}\left(\bar{c}\left(t\right)∥{\bar{c}}_{e}\right)\geq 0,$$

where $\bar{F}\left(t\right)=0$ if and only if $\bar{c}\left(t\right)={\bar{c}}_{e}$ The corresponding inner correction becomes $\tilde{F}\left(\tau \right)=\mathrm{RT}\left[D_{\mathrm{KL}}\left(\bar{c}\left(t\right)+\tilde{c}\left(\tau \right)∥{\bar{c}}_{e}\right)-D_{\mathrm{KL}}\left(\bar{c}\left(t\right)∥{\bar{c}}_{e}\right)\right]$, which can be rewritten as $\tilde{F}\left(\tau \right)=\mathrm{RT}\left[D_{\mathrm{KL}}\left(\bar{c}\left(t\right)+\tilde{c}\left(\tau \right)∥\bar{c}\left(t\right)\right)+\tilde{c}\left(\tau \right)\cdot \ln\frac{\bar{c}\left(t\right)}{{\bar{c}}_{e}}\right]$ in equivalent form. In the following, we verify that the asymptotic expansion of relative entropy remains valid:

(25) $$F^{\epsilon }\left(t\right)=\bar{F}\left(t\right)+\tilde{F}\left(\tau \right)+O\left(\epsilon^{\beta }\right).$$

Thanks to the relation $c_{e}^{\epsilon }={\bar{c}}_{e}+O\left(\epsilon^{\alpha }\right)$, and $c_{e}^{\epsilon }>0,\;{\bar{c}}_{e}>0$, we have $\ln\frac{c^{\epsilon }\left(t\right)}{c_{e}^{\epsilon }}=\ln\frac{c^{\epsilon }\left(t\right)}{{\bar{c}}_{e}}+\ln\frac{{\bar{c}}_{e}}{c_{e}^{\epsilon }}=\ln\frac{c^{\epsilon }\left(t\right)}{{\bar{c}}_{e}}+O\left(\epsilon^{\alpha }\right)$, therefore $D_{\mathrm{KL}}\left(c^{\epsilon }\left(t\right)∥c_{e}^{\epsilon }\right)=D_{\mathrm{KL}}\left(c^{\epsilon }\left(t\right)∥{\bar{c}}_{e}\right)+O\left(\epsilon^{\alpha }\right)$. Applying the uniformly valid approximation of order α to the function $D_{\mathrm{KL}}\left(c^{\epsilon }\left(t\right)∥{\bar{c}}_{e}\right)$, we derive that $D_{\mathrm{KL}}\left(c^{\epsilon }\left(t\right)∥{\bar{c}}_{e}\right)=D_{\mathrm{KL}}\left(\bar{c}\left(t\right)+\tilde{c}\left(\tau \right)∥{\bar{c}}_{e}\right)+O\left(\epsilon^{\beta }\right)$. On the other hand, the matching condition of the relative entropy is guaranteed because of $\lim_{\tau \to \infty }\tilde{F}\left(\tau \right)=0$. This completes the proof.

Following the same philosophy as for the relative entropy, we construct the asymptotic expansion of $f_{d}^{\epsilon }\left(t\right)$ by combining an outer part that uses ${\bar{c}}_{e}$ as reference and an inner correction built from the composite solution. We define the outer free energy dissipation rate as the function evaluated at the outer solution $\bar{c}\left(t\right)$, using the outer steady state ${\bar{c}}_{e}$:

(26) $$\begin{array}{cc} f_{d}^{\epsilon }\left(t\right) & =\bar{f_{d}}\left(t\right)+\tilde{f_{d}}\left(\tau \right)+O\left(\epsilon^{\beta }\right), \end{array}$$

(27) $$\begin{array}{cc} \bar{f_{d}}\left(t\right) & =RT\;R\left(\bar{c}\left(t\right)\right)\cdot \ln\;\left(\frac{R^{+}\left(\bar{c}\left(t\right)\right)\;R^{-}\left({\bar{c}}_{e}\right)}{R^{-}\left(\bar{c}\left(t\right)\right)\;R^{+}\left({\bar{c}}_{e}\right)}\right). \end{array}$$

Here the inner correction is the difference between the free energy dissipation rate evaluated at the composite solution and its value at the outer solution, $\tilde{f_{d}}\left(\tau \right)=RT\;R\left(\bar{c}\left(t\right)+\tilde{c}\left(\tau \right)\right)\cdot \ln\;\left(\frac{R^{+}\left(\bar{c}\left(t\right)+\tilde{c}\left(\tau \right)\right)\;R^{-}\left({\bar{c}}_{e}\right)}{R^{-}\left(\bar{c}\left(t\right)+\tilde{c}\left(\tau \right)\right)\;R^{+}\left({\bar{c}}_{e}\right)}\right)-\bar{f_{d}}\left(t\right)$.

In conclusion, we find that the outer parts of thermodynamic quantities are simply the values of the corresponding thermodynamic functions evaluated at the outer solution $\bar{c}\left(t\right)$. In contrast, the corrected inner parts are not obtained by a naive linearization but are constructed directly from the composite solution: they represent the difference between the thermodynamic function evaluated at the sum of the outer solution and the inner correction, $\bar{c}\left(t\right)+\tilde{c}\left(\tau \right)$, and its value at the outer solution $\bar{c}\left(t\right)$. This construction ensures that the inner corrections capture the nonlinear dependence on the fast variables within the initial layer, vanish as τ→∞, and together with the outer parts provide a uniformly valid approximation for all thermodynamic functions throughout the entire time domain.

## 3. Application to Michaells–Menten Reaction

In this section, we first apply the method of matched asymptotic expansions to the reversible Michaelis--Menten (MM) reaction, and then study the corresponding asympotoic expansions of thermodynamic functions.

The reversible MM reaction is

(28) $$S+E\underset{k_{1}^{-}}{\overset{k_{1}^{+}}{⇌}}C\underset{k_{2}^{-}}{\overset{k_{2}^{+}}{⇌}}P+E,$$

where the initial concentrations are $\left(\left[S\right]\left(0\right),\left[E\right]\left(0\right),\left[C\right]\left(0\right),\left[P\right]\left(0\right)\right)=\left(S_{0},E_{0},0,0\right)$. The total enzyme concentration is conserved, $\left[E\right]+\left[C\right]=E_{0}$, so is the total substrate, $\left[S\right]+\left[C\right]+\left[P\right]=S_{0}$. This motivates us to choose the concentrations of the substrate *S* and the complex *C* as the independent variables. The governing equations are

(29) $$\begin{array}{cc} \frac{d[S]}{dt} & =-k_{1}^{+}\left[S\right]\left[E\right]+k_{1}^{-}\left[C\right],\\ \frac{d[C]}{dt} & =k_{1}^{+}\left[S\right]\left[E\right]-\left(k_{1}^{-}+k_{2}^{+}\right)\left[C\right]+k_{2}^{-}\left[P\right]\left[E\right], \end{array}$$

with the initial condition ${\left(\left[S\right]\left(0\right),\left[C\right]\right)|}_{h=0}=\left(S_{0},0\right)$.

### 3.1. Asymptotic Expansions of MM Reaction

Dimensionless variables. We define the dimensionless variables as

$$x=\frac{\left[S\right]}{S_{0}},\;y=\frac{\left[C\right]}{E_{0}},\;h=k_{1}^{+}E_{0}\;t.$$

Using the conservation laws $\left[E\right]=E_{0}\left(1-y\right)$ and $\left[P\right]=S_{0}\left(1-x\right)-E_{0}y$, the dimensionless equations of the original MM reaction become

(30) $$\begin{array}{cc} \frac{dx}{dh} & =-x(1-y)+\kappa y,\\ \epsilon \frac{dy}{dh} & =(1-\nu )x(1-y)+\nu -(\kappa +\mu +\nu )y-\epsilon \nu y(1-y), \end{array}$$

with the initial condition (x(0),y(0))=(1,0). Here, the small parameter $\epsilon =\frac{E_{0}}{S_{0}}\ll 1$ is the ratio of the initial concentration of enzyme to substrate, and the other three dimensionless parameters are $\kappa =\frac{k_{1}^{-}}{k_{1}^{+}S_{0}},\;\mu =\frac{k_{2}^{+}}{k_{1}^{+}S_{0}},\;\nu =\frac{k_{2}^{-}}{k_{1}^{+}}$.

Leading-order outer solution. For times h=O(1), we seek regular expansions $\bar{x}\left(h\right)={\bar{x}}_{0}\left(h\right)+\epsilon {\bar{x}}_{1}\left(h\right)+\cdots$ and $\bar{y}\left(h\right)={\bar{y}}_{0}\left(h\right)+\epsilon {\bar{y}}_{1}\left(h\right)+\cdots$. The leading-order outer solutions $\left({\bar{x}}_{0}\left(h\right),{\bar{y}}_{0}\left(h\right)\right)$ satisfy the differential-algebraic system obtained by inserting the above regular expansions into Equation (30) and collecting the O(1) terms:

(31a) $$\begin{array}{cc} 0 & =\left(1-\nu \right){\bar{x}}_{0}\left(1-{\bar{y}}_{0}\right)+\nu -\left(\kappa +\mu +\nu \right){\bar{y}}_{0}, \end{array}$$

(31b) $$\begin{array}{cc} \frac{d{\bar{x}}_{0}}{dh} & =-{\bar{x}}_{0}+\left({\bar{x}}_{0}+\kappa \right){\bar{y}}_{0},\;{\bar{x}}_{0}\left(0\right)=1. \end{array}$$

Equation (31a) gives the dimensionless complex concentration ${\bar{y}}_{0}\left(h\right)$ in terms of the substrate concentration ${\bar{x}}_{0}\left(h\right)$:

(32) $${\bar{y}}_{0}=\frac{\left(1-\nu \right){\bar{x}}_{0}+\nu }{\left(1-\nu \right){\bar{x}}_{0}+\left(\kappa +\mu +\nu \right)}\equiv \phi \left({\bar{x}}_{0}\right),$$

which is called the quasi-steady-state relation. Substituting this into Equation (31b) yields a closed differential equation for ${\bar{x}}_{0}\left(h\right)$:

(33) $$\frac{d{\bar{x}}_{0}}{dh}=\frac{\kappa \nu -\left(\mu +\kappa \nu \right){\bar{x}}_{0}}{\left(1-\nu \right){\bar{x}}_{0}+\left(\kappa +\mu +\nu \right)},\;{\bar{x}}_{0}\left(0\right)=1.$$

Separating variables and integrating this equation directly yields an implicit relation for the outer solution ${\bar{x}}_{0}\left(h\right)$:

(34) $$h+J=-\frac{1-\nu }{\mu +\kappa \nu }\;{\bar{x}}_{0}-\frac{(\kappa +\mu +\nu )(\mu +\kappa \nu )+(1-\nu )\kappa \nu }{{\left(\mu +\kappa \nu \right)}^{2}}\;\ln\left|\left(\mu +\kappa \nu \right){\bar{x}}_{0}-\kappa \nu \right|,$$

where the integration constant *J* will be determined later by the matching condition. Here is a remark on the dimensionless scheme.

**Remark** **2.** *The small parameter $\epsilon =E_{0}/S_{0}$ reflects the typical situation in which the enzyme concentration is much lower than the substrate concentration. The time scale $h=k_{1}^{+}E_{0}t$ is based on the fast enzyme-substrate binding step, which dominates the initial layer. The three dimensionless parameters κ,μ and ν capture the relative rates of the reverse binding, forward product formation, and reverse product formation, respectively. When ν=0 the system degenerates to the classic irreversible MM model, and the outer Equation (33) simplifies to $\frac{d{\bar{x}}_{0}}{dh}=-\frac{\mu {\bar{x}}_{0}}{{\bar{x}}_{0}+\kappa +\mu }$, in agreement with Equation (5) of [2]. Hence, the present scaling is both mathematically convenient and physically natural.*

Leading-order inner solution. Introduce the fast time variable τ=h/ϵ and set $\left(x^{\epsilon }\left(\epsilon \tau \right),y^{\epsilon }\left(\epsilon \tau \right)\right)=\left({\hat{x}}^{\epsilon }\left(\tau \right),{\hat{y}}^{\epsilon }\left(\tau \right)\right)$. The inner equations are

$$\begin{array}{cc} \frac{d{\hat{x}}^{\epsilon }}{d\tau } & =\epsilon \left[-{\hat{x}}^{\epsilon }+\left({\hat{x}}^{\epsilon }+\kappa \right){\hat{y}}^{\epsilon }\right],\\ \frac{d{\hat{y}}^{\epsilon }}{d\tau } & =\left(1-\nu \right){\hat{x}}^{\epsilon }\left(1-{\hat{y}}^{\epsilon }\right)+\nu -\left(\kappa +\mu +\nu \right){\hat{y}}^{\epsilon }-\epsilon \nu {\hat{y}}^{\epsilon }\left(1-{\hat{y}}^{\epsilon }\right), \end{array}$$

with the initial condition $({\hat{x}}^{\epsilon }\left(0\right),{\hat{y}}^{\epsilon }\left(0\right))=\left(1,0\right)$. The expansions of $\left({\hat{x}}^{\epsilon }\left(\tau \right),{\hat{y}}^{\epsilon }\left(\tau \right)\right)$ give, in the leading order,

$$\frac{d{\hat{x}}_{0}}{d\tau }=0,\;\frac{d{\hat{y}}_{0}}{d\tau }=\left(1-\nu \right){\hat{x}}_{0}\left(1-{\hat{y}}_{0}\right)+\nu -\left(\kappa +\mu +\nu \right){\hat{y}}_{0}.$$

We obtain ${\hat{x}}_{0}\left(\tau \right)\equiv 1$ and $\frac{d{\hat{y}}_{0}}{d\tau }=1-B{\hat{y}}_{0}$ with B=1+κ+μ. Hence,

(35) $${\hat{y}}_{0}\left(\tau \right)=\frac{1}{B}\left(1-e^{-B\tau }\right).$$

Matching. Matching requires that the inner solution as τ→∞ agrees with the outer solution as h→0. To be specific, from Equation (35), $\lim_{\tau \to \infty }{\hat{y}}_{0}\left(\tau \right)=\frac{1}{B}$. The quasi-steady-state relation (32) with ${\bar{x}}_{0}\left(0\right)=1$ gives the same value, ${\bar{y}}_{0}\left(0\right)=\frac{(1-\nu )\cdot 1+\nu }{\left(1-\nu )\cdot 1+(\kappa +\mu +\nu \right)}=\frac{1}{B}$. Thus, the matching conditions are ${\bar{x}}_{0}\left(0\right)=1,\;{\bar{y}}_{0}\left(0\right)=\frac{1}{B}$. These conditions determine the integration constant by substituting h=0 and ${\bar{x}}_{0}\left(0\right)=1$ into Equation (34) as, $J=-\frac{1-\nu }{\mu +\kappa \nu }-\frac{(\kappa +\mu +\nu )(\mu +\kappa \nu )+(1-\nu )\kappa \nu }{{\left(\mu +\kappa \nu \right)}^{2}}\;\ln\left|\mu \right|$. Thus, the following equation provides the leading-order outer solution ${\bar{x}}_{0}\left(h\right)$ in an implicit form:

(36) $$h+\frac{1-\nu }{\mu +\kappa \nu }\left({\bar{x}}_{0}-1\right)+\frac{(\kappa +\mu +\nu )(\mu +\kappa \nu )+(1-\nu )\kappa \nu }{{\left(\mu +\kappa \nu \right)}^{2}}\ln\left|{\bar{x}}_{0}+\frac{\kappa \nu }{\mu }\left({\bar{x}}_{0}-1\right)\right|=0.$$

At this stage, a uniformly valid leading-order composite solution is obtained by adding the inner and outer solutions and subtracting their common limit. The leading-order composite solutions for the dimensionless substrate concentration and the complex concentration are

(37) $$\begin{array}{cc} \; & x^{\epsilon }\left(h\right)={\bar{x}}_{0}\left(h\right)+O\left(\epsilon \right),\\ \; & y^{\epsilon }\left(h\right)={\bar{y}}_{0}\left(h\right)+{\tilde{y}}_{0}\left(\tau \right)+O\left(\epsilon \right), \end{array}$$

where the outer solution ${\bar{x}}_{0}\left(h\right)$ is determined by Equation (36), and ${\bar{y}}_{0}\left(h\right)$ by the quasi-steady-state relation in Equation (32), the corrected inner solution is ${\tilde{y}}_{0}\left(\tau \right)={\hat{y}}_{0}\left(\tau \right)-{\bar{y}}_{0}\left(0\right)=-\frac{1}{B}\;e^{-B\tau }$.

Although the leading-order composite solution provides a good approximation, it is unable to reveal the higher-order asymptotic properties of the MM reaction.

First-order expansions. To uncover the higher-order properties, we now extend the asymptotic expansions (37) to the first order in ϵ as

(38) $$\begin{array}{cc} \; & x^{\epsilon }\left(h\right)={\bar{x}}_{0}\left(h\right)+\epsilon {\bar{x}}_{1}\left(h\right)+\epsilon {\tilde{x}}_{1}\left(\tau \right)+O\left(\epsilon^{2}\right),\\ \; & y^{\epsilon }\left(h\right)={\bar{y}}_{0}\left(h\right)+\epsilon {\bar{y}}_{1}\left(h\right)+{\tilde{y}}_{0}\left(\tau \right)+\epsilon {\tilde{y}}_{1}\left(\tau \right)+O\left(\epsilon^{2}\right). \end{array}$$

The detailed derivation of the first-order equations is provided in Appendix A; merely the final results are presented here.

The outer solutions $\left({\bar{x}}_{1}\left(h\right),\;{\bar{y}}_{1}\left(h\right)\right)$ satisfy:

(39a) $$\begin{array}{cc} \frac{d{\bar{x}}_{1}}{dh} & =\left({\bar{y}}_{0}-1\right){\bar{x}}_{1}+\left({\bar{x}}_{0}+\kappa \right){\bar{y}}_{1}, \end{array}$$

(39b) $$\begin{array}{cc} \left[\left(1-\nu \right){\bar{x}}_{0}+\kappa +\mu +\nu \right]{\bar{y}}_{1} & =\left(1-\nu \right)\left(1-{\bar{y}}_{0}\right){\bar{x}}_{1}-\frac{d{\bar{y}}_{0}}{dh}-\nu {\bar{y}}_{0}\left(1-{\bar{y}}_{0}\right), \end{array}$$

where the initial value ${\bar{x}}_{1}\left(0\right)=-\frac{1+\kappa }{B^{2}}$ is determined by matching with the inner layer. The first-order corrected inner solutions are given explicitly by

(40) $${\tilde{x}}_{1}\left(\tau \right)=\frac{1+\kappa }{B^{2}}\;e^{-B\tau },$$

and

(41) $${\tilde{y}}_{1}\left(\tau \right)=\left[-\frac{(1-\nu )\mu }{2B^{2}}\;\tau^{2}+\frac{\left(B-2)(1+\kappa +\nu \mu \right)}{B^{3}}\;\tau -\frac{1+\kappa +\nu \mu }{B^{4}}\;e^{-B\tau }+K\right]e^{-B\tau },$$

where the integration constant $K=\frac{1}{B^{2}}-\frac{\mu (1-\nu )(2B-1)}{B^{4}}$.

Collecting the outer and the corrected inner contributions, the uniformly valid first-order asymptotic solution on the whole interval h∈[0,T] is obtained in Equation (38). Here $\left({\bar{x}}_{1},{\bar{y}}_{1}\right)$ are solved from (39a) and (39b), and $\left({\tilde{x}}_{1},{\tilde{y}}_{1}\right)$ are given analytically in Equations (40) and (41) above. All correction functions vanish exponentially as τ→∞, ensuring that the composite solution matches the outer solution uniformly outside the initial layer. The positivity of the outer solution and the associated expansion for the product concentration are treated in Appendix A.2.

The MM reaction has been extensively studied through various frameworks, see, e.g., [5,6,8]. Heineken et al. [8] reduced the irreversible MM reaction by QSSA, whose result is equivalent to the leading-order outer solution based on singular perturbation theory in the irreversible limit, $k_{2}^{-}=0$. Gorban and Shahzad [6] showed that QSSA and quasiequilibrium hypothesis together lead to thermodynamically consistent kinetic laws. In contrast to these works, the present study considers the fully reversible MM reaction and derives explicit matched asymptotic expansions for both the kinetics and the associated nonequilibrium thermodynamics.

### 3.2. Thermodynamics for MM Reaction

For the MM reaction, we denote the time-dependent concentration vector as $c\left(h\right)={\left(\left[S\right],\left[E\right],\left[C\right],\left[P\right]\right)}^{T}$. In terms of the dimensionless variables $x^{\epsilon }\left(h\right)$ and $y^{\epsilon }\left(h\right)$, the vector c(h) is rewritten as

(42) $$c\left(h\right)={\left(S_{0}x^{\epsilon },E_{0}\left(1-y^{\epsilon }\right),E_{0}y^{\epsilon },S_{0}\left(1-x^{\epsilon }\right)-E_{0}y^{\epsilon }\right)}^{T}.$$

Correspondingly, the steady state $c_{e}^{\epsilon }$ of the original MM kinetics (30) and the steady state ${\bar{c}}_{e}$ of the reduced kinetics (31a) and (31b) are derived in the Appendix B.

By choosing the enthalpy as in Equation (10), the entropy as in Equation (12), the entropy production rate as in Equation (13), and the relative entropy as in Equation (14), we have the original thermodynamic functions:

(43a) $$\begin{array}{cc} H^{\epsilon }\left(h\right) & =S_{0}x^{\epsilon }\left(h_{S}^{\circ }-h_{P}^{\circ }\right)+E_{0}y^{\epsilon }\left(h_{C}^{\circ }-h_{E}^{\circ }-h_{P}^{\circ }\right)+\left(E_{0}h_{E}^{\circ }+S_{0}h_{P}^{\circ }\right),\\ {\mathrm{Ent}}^{\epsilon }\left(h\right) & =-R[S_{0}x^{\epsilon }\left(\ln\left(S_{0}x^{\epsilon }\right)-1\right)+E_{0}\left(1-y^{\epsilon }\right)\left(\ln\left(E_{0}\left(1-y^{\epsilon }\right)\right)-1\right)\\ \; & \;+E_{0}y^{\epsilon }\left(\ln\left(E_{0}y^{\epsilon }\right)-1\right)+\left(S_{0}\left(1-x^{\epsilon }\right)-E_{0}y^{\epsilon }\right)\left(\ln\left(S_{0}\left(1-x^{\epsilon }\right)-E_{0}y^{\epsilon }\right)-1\right)] \end{array}$$

(43b) $$\begin{array}{cc} \; & \;+S_{0}x^{\epsilon }s_{S}^{\circ }+E_{0}\left(1-y^{\epsilon }\right)s_{E}^{\circ }+E_{0}y^{\epsilon }s_{C}^{\circ }+\left(S_{0}\left(1-x^{\epsilon }\right)-E_{0}y^{\epsilon }\right)s_{P}^{\circ },\\ {\mathrm{epr}}^{\epsilon }\left(h\right) & =R[\left(k_{1}^{+}S_{0}E_{0}x^{\epsilon }\left(1-y^{\epsilon }\right)-k_{1}^{-}E_{0}y^{\epsilon }\right)\ln\frac{k_{1}^{+}S_{0}x^{\epsilon }\left(1-y^{\epsilon }\right)}{k_{1}^{-}y^{\epsilon }} \end{array}$$

(43c) $$\begin{array}{cc} \; & \;+\left(k_{2}^{+}E_{0}y^{\epsilon }-k_{2}^{-}E_{0}\left(1-y^{\epsilon }\right)\left(S_{0}\left(1-x^{\epsilon }\right)-E_{0}y^{\epsilon }\right)\right)\ln\frac{k_{2}^{+}y^{\epsilon }}{k_{2}^{-}\left(1-y^{\epsilon }\right)\left(S_{0}\left(1-x^{\epsilon }\right)-E_{0}y^{\epsilon }\right)}],\\ F^{\epsilon }\left(h\right) & =RT[S_{0}x^{\epsilon }\ln\frac{x^{\epsilon }}{x_{e}^{\epsilon }}+E_{0}\left(1-y^{\epsilon }\right)\ln\frac{1-y^{\epsilon }}{1-y_{e}^{\epsilon }}+E_{0}y^{\epsilon }\ln\frac{y^{\epsilon }}{y_{e}^{\epsilon }} \end{array}$$

(43d) $$\begin{array}{cc} \; & \;+\left(S_{0}\left(1-x^{\epsilon }\right)-E_{0}y^{\epsilon }\right)\ln\frac{S_{0}\left(1-x^{\epsilon }\right)-E_{0}y^{\epsilon }}{S_{0}\left(1-x_{e}^{\epsilon }\right)-E_{0}y_{e}^{\epsilon }}+E_{0}\left(y^{\epsilon }-y_{e}^{\epsilon }\right)]. \end{array}$$

Moreover, the entropy flow rate is determined by the entropy balance equation, $J_{f}^{\epsilon }\left(h\right)=\frac{d}{dh}{\mathrm{Ent}}^{\epsilon }\left(h\right)-{\mathrm{epr}}^{\epsilon }\left(h\right)$.

### 3.3. Asymptotic Expansions of Thermodynamics for MM Reaction

According to the asymptotic expansions of thermodynamics for general CRNs in Section 2.4, we obtain the corresponding results for the MM reaction (28). The asymptotic thermodynamics can be obtained directly based on Equations (18), (43a), (43b), (43c) and (43d).

**Figure 1:**
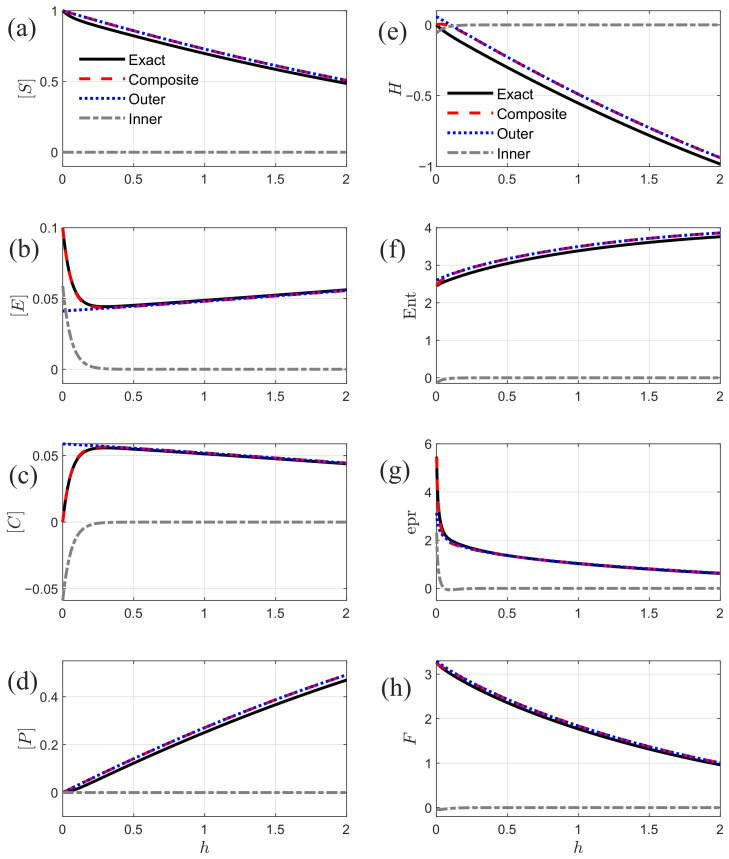
Leading-order kinetics and thermodynamics for the MM reaction. (**a**–**d**) Concentrations: substrate [S] (**a**), enzyme [E] (**b**), complex [C] (*c*), and product [P] (**d**). (**e**–**h**) Thermodynamics: enthalpy H(h) (**e**), entropy Ent(h) (**f**), entropy production rate epr(h) (**g**), and relative entropy F(h) (**h**). In each panel, the exact solution (black solid line) is compared with its composite approximation of leading order (red dashed line), the outer part (blue dotted line) and the inner correction (grey dash-dotted line) are also plotted. All quantities are shown over h∈[0,2] to highlight the multiscale structure. Parameters: $S_{0}=1.0$, $E_{0}=0.1$, $k_{1}^{+}=10$, $k_{1}^{-}=2$, $k_{2}^{+}=5$, $k_{2}^{-}=1$ (hence ϵ=0.1, κ=0.2, μ=0.5, ν=0.1); standard thermodynamic data: $h_{S}=h_{E}=0$, $h_{C}=-1$, $h_{P}=-2$, $s_{S}=s_{E}=1$, $s_{C}=\ln5+1$, $s_{P}=2\ln5-1$, R=T=1.

The numerical validation in Figure 1 clearly demonstrates the effectiveness of the proposed leading-order asymptotic expansion. For all kinetic and thermodynamic quantities, including the concentrations in Figure 1a–d, enthalpy, entropy, entropy production rate, and relative entropy in Figure 1e–h, the leading-order composite approximation fits the exact solution well, including the initial layer. The outer solution fails to capture the rapid transient and the inner correction alone vanishes outside the initial layer. This uniform validity across different thermodynamic quantities, linear or nonlinear, demonstrates that the construction provides a general and physically consistent method for multiscale thermodynamics. Consequently, the present approach bridges the gap between the singular perturbation theory of reaction kinetics and nonequilibrium thermodynamics.

Figure 1c depicts the time-dependent trajectory of the fast variable $\left[C\right]=E_{0}y\left(h\right)$. The outer part $E_{0}{\bar{y}}_{0}\left(h\right)$ captures the long-term trend, while the corrected inner part $E_{0}{\tilde{y}}_{0}\left(h/\epsilon \right)$, approaching 0 quickly for h≥0.2, reflects rapid changes within the initial layer. Similarly, as shown in Figure 1g, both the full and the composite entropy production rates, ${\mathrm{epr}}^{\epsilon }\left(h\right)$ and $\left[\bar{\mathrm{epr}}\left(h\right)+\tilde{\mathrm{epr}}\left(h/\epsilon \right)\right]$, change rapidly in the initial layer and then decrease to a small positive value, as a manifestation of the second law of thermodynamics. The monotonic decreasing property of the relative entropy F(h) illustrated in Figure 1h further supports the above findings.

The mean absolute error (MAE) and the maximum absolute error ($L_{\infty }$ error) are employed to assess the accuracy of the proposed asymptotic approximations quantitatively. For a time-dependent quantity $Q^{\epsilon }\left(h\right)$ and its approximation $Q_{\mathrm{comp}}^{\epsilon }\left(h\right)$, these are defined over a discrete set of *N* time points ${\left\{h_{k}\right\}}_{k=1}^{N}$ as

$$\mathrm{MAE}\left(Q\right)=\frac{1}{N}\sum_{k=1}^{N}\left|Q^{\epsilon }\left(h_{k}\right)-Q_{\mathrm{comp}}^{\epsilon }\left(h_{k}\right)\right|,\;L_{\infty }\left(Q\right)=\max_{1\leq k\leq N}\left|Q^{\epsilon }\left(h_{k}\right)-Q_{\mathrm{comp}}^{\epsilon }\left(h_{k}\right)\right|.$$

The MAE measures the average global accuracy over the entire time interval, while the $L_{\infty }$ error captures the worst-case pointwise deviation, which is sensitive to inaccuracies within the initial layer. The evaluation is performed over a sufficiently long time domain $h\in (0,h_{\max}]$, where the initial singular point h=0 is excluded to avoid the non-physical divergence of the entropy, entropy production rate, and relative entropy due to ${\left[C\right]|}_{h=0}{=\left[P\right]|}_{h=0}=0$.

**Figure 2:**
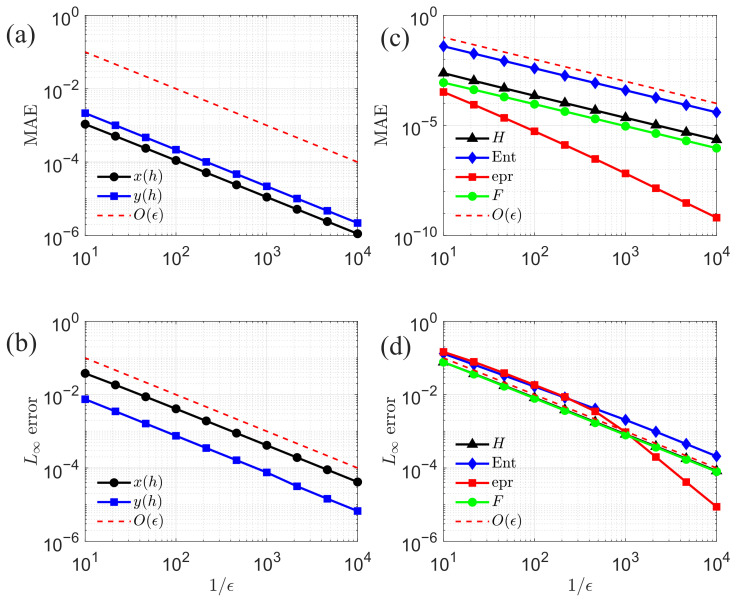
Error analysis of leading-order kinetics and thermodynamics as a function of ϵ for the MM reaction. (**a**,**b**) Kinetics. The MAEs (**a**) and $L_{\infty }$ errors (**b**) of x(h) (black circles) and y(h) (blue squares) are shown. (**c**,**d**) Thermodynamics. The MAEs (**c**) and $L_{\infty }$ errors (**d**) of enthalpy H(h) (black triangles), entropy Ent(h) (blue diamonds), entropy production rate epr(h) (red squares), and relative entropy F(h) (green circles) are presented. In each panel, the red dashed line, error=ϵ, indicates the theoretical first-order convergence order O(ϵ), demonstrating that the MAEs of the composite approximation scale linearly with ϵ. Both the MAE and $L_{\infty }$ errors are evaluated over h∈(0,200]. All the parameters are the same as those in Figure 1, except that $E_{0}$ is redefined as $\epsilon S_{0}$ with a range of $\epsilon \in [10^{-4},10^{-1}]$.

As shown in the log-log plots of Figure 2, the leading-order composite approximations for both kinetic and thermodynamic quantities exhibit at least first-order convergence with respect to ϵ. In Figure 2a,b, the MAEs and $L_{\infty }$ errors for the dimensionless concentrations x(h) and y(h) decrease linearly with ϵ, closely following the theoretical reference line error=ϵ. For the thermodynamic quantities in Figure 2c,d, the MAEs and $L_{\infty }$ errors of enthalpy H(h), entropy Ent(h), and relative entropy F(h) also scale as O(ϵ). In particular, the entropy production rate epr(h) achieves even higher accuracy. Its MAE decreases with a fitted slope of 1.91 in the log-log plot, corresponding to an effective convergence order close to $O(\epsilon^{2})$. The $L_{\infty }$ error of epr(h) shows a piecewise scaling behavior. For $\epsilon \in [2.2\times 10^{-3},10^{-1}]$ the slope is approximately 1, while for the smaller values $\epsilon \in [10^{-4},2.2\times 10^{-3})$ the slope increases to 1.95, again approaching a second-order convergence $O(\epsilon^{2})$.

For results on first-order composite solutions, readers are referred to Appendix C for details.

The convergence orders of the reversible MM reaction (28) are summarized as follows. Let $c^{\epsilon }\left(h\right)=\bar{c}\left(h\right)+\tilde{c}\left(\tau \right)+O\left(\epsilon^{\alpha }\right)$ be the composite expansion of the concentrations with α∈{1,2}, where $\tilde{c}\left(\tau \right)\to 0$ exponentially as τ→∞. We observe that $\bar{c}\left(h\right)$ and $\bar{c}\left(h\right)+\tilde{c}\left(\tau \right)$ remain within a compact subset of ${\left(0,\infty \right)}^{N}$ for all $h\geq h_{0}>0$, i.e., strictly positive after an arbitrarily short initial transient. Then for small ϵ and for all $h\geq h_{0}>0$:

- For the enthalpy, $H^{\epsilon }\left(h\right)=\bar{H}\left(h\right)+\tilde{H}\left(\tau \right)+O\left(\epsilon^{\alpha }\right)$;
- For the entropy and the relative entropy, ${\mathrm{Ent}}^{\epsilon }\left(h\right)=\bar{\mathrm{Ent}}\left(h\right)+\tilde{\mathrm{Ent}}\left(\tau \right)+O\left(\epsilon \right),\;F^{\epsilon }\left(h\right)=\bar{F}\left(h\right)+\tilde{F}\left(\tau \right)+O\left(\epsilon \right)$;
- For the entropy production rate, ${\mathrm{epr}}^{\epsilon }\left(h\right)=\bar{\mathrm{epr}}\left(h\right)+\tilde{\mathrm{epr}}\left(\tau \right)+O\left(\epsilon^{\alpha }\right)$.

## 4. Conclusions

In this work, we have presented a general framework that integrates singular perturbation theory with nonequilibrium thermodynamics for CRNs possessing a clear separation of time scales. By applying the additive composite expansion—originally developed for the concentrations—directly to the thermodynamic functionals, we have obtained a uniformly valid approximation that naturally splits into a slow-varying outer part and a fast-varying corrected inner part. This approach avoids any linearization of the thermodynamic quantities and captures the nonlinear behavior inside the inner layer.

For a general reversible CRN with fast–slow kinetics (5), we have derived the composite expansions for the first and second laws of thermodynamics in Section 2.4, including enthalpy, entropy, entropy production rate, and relative entropy. The outer parts of these quantities depend only on the outer concentration fields and capture the long-time trend, while the inner corrections decay to zero as the fast time variable tends to infinity. Thus, the two-scale character of the thermodynamics is revealed.

The convergence order of the thermodynamic approximations relative to the exact solutions is determined by the local Lipschitz properties of the respective functions. The enthalpy, being a linear function of concentrations, retains the same convergence order as the kinetic variables. In contrast, the entropy, relative entropy, and entropy production rate involve logarithmic terms that cause their gradients to diverge when any concentration approaches zero. We have shown that this logarithmic degeneracy reduces the theoretical convergence order of these three quantities by one relative to the concentration expansion.

The general framework has been fully validated on the reversible MM reaction as a representative example, for which both leading-order and first-order matched asymptotic expansions of the kinetics have been obtained analytically. By introducing an independent expansion for the product [P], the positivity of all the outer and composite concentrations is guaranteed throughout the time domain h>0, a prerequisite for the thermodynamic functions to be well defined. Numerical simulations confirm that the composite thermodynamic approximations are uniformly accurate over the whole time interval, including the initial layer. The numerical error analysis reveals that the entropy production rate converges with a higher order than theoretical predictions. Although the entropy and the relative entropy do not improve beyond O(ϵ) even with the first-order kinetic expansion, the entropy production rate achieves an $O(\epsilon^{2})$ accuracy.

Taken together, the results demonstrate that the composite expansion provides a rigorous and physically consistent tool for analyzing energy and entropy balances in multiscale CRNs. The clear separation between outer and inner contributions, together with the quantitative convergence orders, enables one to identify the dominant thermodynamic processes on each time scale and to construct reduced models with guaranteed accuracy.

Within our framework, this method can be directly applied to more complex CRNs with fast–slow structure, such as the protein phosphorylation-dephosphorylation cycle (PdPC) [29] and the signal transduction in apoptosis [4]. In practice, the outer concentration alone suffices to capture the long-time trend of thermodynamic functions. If the inner concentration is also available from experimental observation, the changes in the thermodynamic quantities inside the initial layer can be restored, and the theoretical convergence order can be ensured. Future work will extend the method to CRNs with spatial multiscality, such as the reaction–diffusion systems.

## Figures and Tables

**Figure A1:**
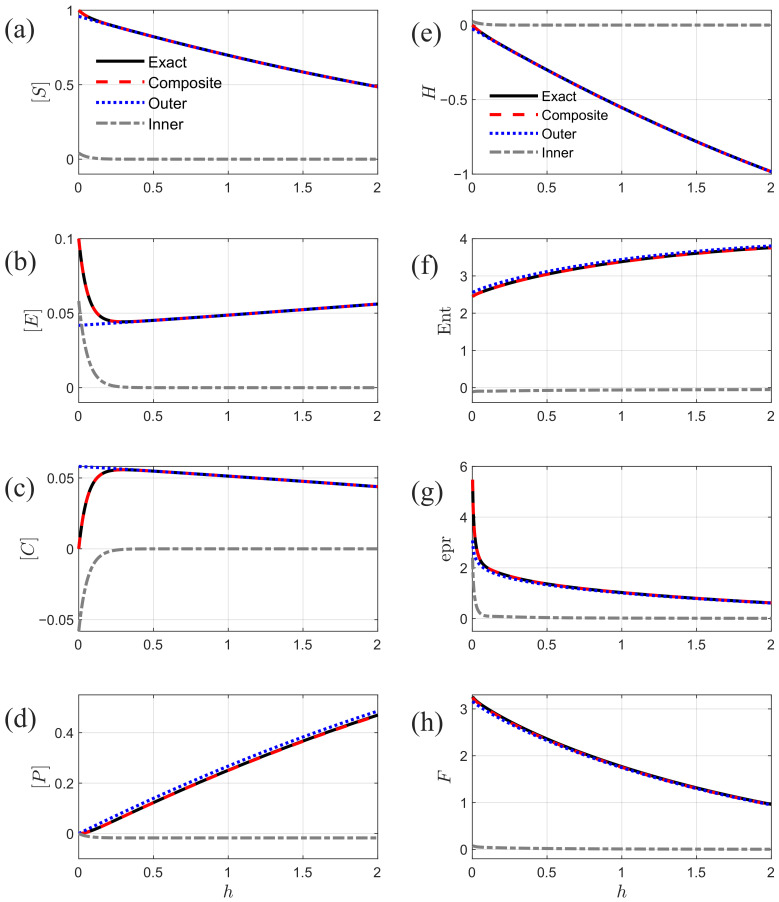
First-order kinetics and thermodynamics for the MM reaction. (**a**–**d**) Concentrations. (**e**–**h**) Thermodynamics. In each panel, the exact solution (black solid line) is compared with the composite approximation of first order (red dashed line), the outer part (blue dotted line) and the inner correction (grey dash-dotted line) are also shown. All the parameters are the same as those in Figure 1.

**Figure A2:**
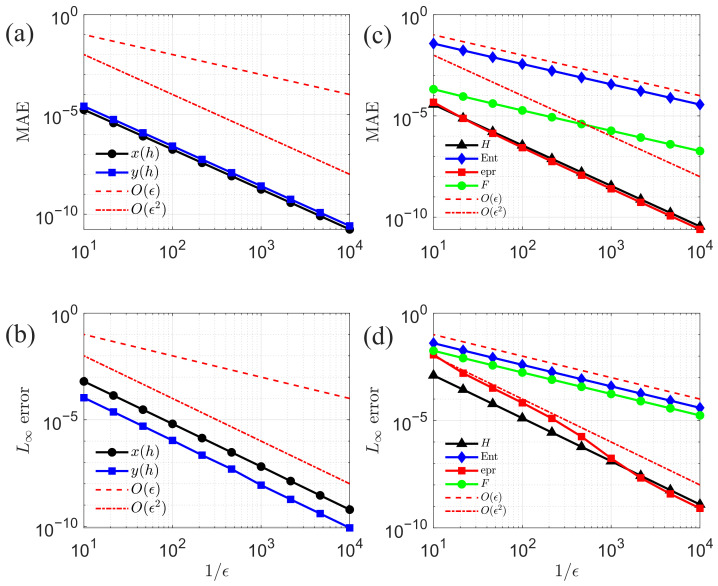
Error analysis of first-order kinetics and thermodynamics as a function of ϵ for the MM reaction. (**a**,**b**) Kinetics. (**c**,**d**) Thermodynamics. In each panel, the red dashed line error=ϵ and the red dashed-dotted line $\mathrm{error}=\epsilon^{2}$ indicate the first-order and second-order convergence rates respectively. Both the MAE and $L_{\infty }$ errors are evaluated over h∈(0,200]. All the parameters are the same as those in Figure 1, except that $E_{0}$ is redefined as $\epsilon S_{0}$ with a range of $\epsilon \in [10^{-4},10^{-1}]$.

## Data Availability

The original contributions presented in this study are included in the article. Further inquiries can be directed to the corresponding authors.

## Abbreviations

The following abbreviations are used in this manuscript:

CRN	Chemical Reaction Network
QSSA	Quasi-Steady-State Approximation
PEA	Partial Equilibrium Approximation
ODE	Ordinary Differential Equation
MM	Michaelis–Menten
MAE	Mean Absolute Error
KL divergence	Kullback–Leibler divergence

## Appendix A. Details in First-Order Asymptotic Expansions

### Appendix A.1. Derivation of First-Order Outer and Inner Equations

We provide a detailed derivation of the first-order outer Equations (39a) and (39b) and the corrected inner equations, together with their solutions.

First-order outer equations. Insert the regular expansions $\bar{x}\left(h\right)={\bar{x}}_{0}\left(h\right)+\epsilon {\bar{x}}_{1}\left(h\right)+O\left(\epsilon^{2}\right),\;\bar{y}\left(h\right)={\bar{y}}_{0}\left(h\right)+\epsilon {\bar{y}}_{1}\left(h\right)+O\left(\epsilon^{2}\right)$ into the full dimensionless system

$$\begin{array}{cc} \frac{dx}{dh} & =F(x,y)=-x(1-y)+\kappa y,\\ \epsilon \frac{dy}{dh} & =G(x,y)-\epsilon \nu y(1-y),\;G(x,y)=(1-\nu )x(1-y)+\nu -(\kappa +\mu +\nu )y. \end{array}$$

Perform a Taylor expansion of the right side of the above equation around the point $\left({\bar{x}}_{0},{\bar{y}}_{0}\right)$. For the first equation, we obtain

$$\frac{d{\bar{x}}_{0}}{dh}+\epsilon \frac{d{\bar{x}}_{1}}{dh}=F\left({\bar{x}}_{0},{\bar{y}}_{0}\right)+\epsilon \left[F_{x}\left({\bar{x}}_{0},{\bar{y}}_{0}\right){\bar{x}}_{1}+F_{y}\left({\bar{x}}_{0},{\bar{y}}_{0}\right){\bar{y}}_{1}\right]+O\left(\epsilon^{2}\right),$$

with $F_{x}={\bar{y}}_{0}-1$ and $F_{y}={\bar{x}}_{0}+\kappa$. For the second equation, we obtain

$$\epsilon \frac{d{\bar{y}}_{0}}{dh}+\epsilon^{2}\frac{d{\bar{y}}_{1}}{dh}=G\left({\bar{x}}_{0},{\bar{y}}_{0}\right)+\epsilon \left[G_{x}\left({\bar{x}}_{0},{\bar{y}}_{0}\right){\bar{x}}_{1}+G_{y}\left({\bar{x}}_{0},{\bar{y}}_{0}\right){\bar{y}}_{1}-\nu {\bar{y}}_{0}\left(1-{\bar{y}}_{0}\right)\right]+O\left(\epsilon^{2}\right),$$

where $G_{x}=\left(1-\nu \right)\left(1-{\bar{y}}_{0}\right)$ and $G_{y}=-\left[\left(1-\nu \right){\bar{x}}_{0}+\kappa +\mu +\nu \right]$.

Equating powers of ϵ gives at O(1), $\frac{d{\bar{x}}_{0}}{dh}=F\left({\bar{x}}_{0},{\bar{y}}_{0}\right),\;0=G\left({\bar{x}}_{0},{\bar{y}}_{0}\right)$, we have the zeroth-order Equations (31a) and (31b) in the main text. At O(ϵ), the first equation yields directly

$$\frac{d{\bar{x}}_{1}}{dh}=F_{x}{\bar{x}}_{1}+F_{y}{\bar{y}}_{1}=\left({\bar{y}}_{0}-1\right){\bar{x}}_{1}+\left({\bar{x}}_{0}+\kappa \right){\bar{y}}_{1},$$

which is (39a). The second equation gives

$$\frac{d{\bar{y}}_{0}}{dh}=G_{x}{\bar{x}}_{1}+G_{y}{\bar{y}}_{1}-\nu {\bar{y}}_{0}\left(1-{\bar{y}}_{0}\right),$$

which is (39b). Thus the first-order outer terms satisfy the linear system (39a) and (39b).

By recalling the fact that $d{\bar{y}}_{0}/dh=d\phi ({\bar{x}}_{0})/dh=\phi^{\prime }\left({\bar{x}}_{0}\right)\cdot d{\bar{x}}_{0}/dh$, it can be deduced that ${\bar{y}}_{1}\left(h\right)$ is decoupled from ${\bar{x}}_{1}\left(h\right)$, and ${\bar{y}}_{1}\left(h\right)$ is given by the algebraic equation involving ${\bar{x}}_{1}\left(h\right)$ and lower-order terms as

$${\bar{y}}_{1}\left(h\right)=\frac{1}{D}\left[\left(1-\nu \right)\left(1-{\bar{y}}_{0}\right){\bar{x}}_{1}-\nu {\bar{y}}_{0}\left(1-{\bar{y}}_{0}\right)-\phi^{\prime }\left({\bar{x}}_{0}\right)\frac{d{\bar{x}}_{0}}{dh}\right],$$

where $D=\left(1-\nu \right){\bar{x}}_{0}\left(h\right)+\kappa +\mu +\nu$. Therefore, we have

(A1) $$\frac{d{\bar{x}}_{1}}{dh}=-\frac{\left(\kappa +\mu \right)\left[\mu +\nu (1+\kappa )\right]}{D^{2}}\;{\bar{x}}_{1}\;-\;\frac{\left({\bar{x}}_{0}+\kappa \right)\left(\kappa +\mu \right)}{D^{3}}\left[\nu \left(\left(1-\nu \right){\bar{x}}_{0}+\nu \right)+\left(1-\nu \right)\frac{d{\bar{x}}_{0}}{dh}\right].$$

The initial condition for ${\bar{x}}_{1}$ is fixed by matching with the initial layer, which will be specified below.

First-order corrected inner equations. Recall the decompositions of the composite solutions $x^{\epsilon }\left(t\right)=\bar{x}\left(t\right)+\tilde{x}\left(\tau \right)+\cdots$ and $y^{\epsilon }\left(t\right)=\bar{y}\left(t\right)+\tilde{y}\left(\tau \right)+\cdots$ with τ=h/ϵ. Substituting these decompositions into Equation (30) and subtracting the outer equations satisfied by $\left(\bar{x}\left(t\right),\bar{y}\left(t\right)\right)$ yield the exact evolution for the corrected inner solutions:

(A2a) $$\begin{array}{cc} \frac{1}{\epsilon }\frac{d\tilde{x}}{d\tau } & =F\left(\bar{x}+\tilde{x},\bar{y}+\tilde{y}\right)-F\left(\bar{x},\bar{y}\right), \end{array}$$

(A2b) $$\begin{array}{cc} \frac{d\tilde{y}}{d\tau } & =\left[G\left(\bar{x}+\tilde{x},\bar{y}+\tilde{y}\right)-G\left(\bar{x},\bar{y}\right)\right]-\epsilon \nu \left[\left(\bar{y}+\tilde{y}\right)\left(1-\bar{y}-\tilde{y}\right)-\bar{y}\left(1-\bar{y}\right)\right]. \end{array}$$

Expanding the right-hand sides using the explicit forms of *F* and *G*:

$$\begin{array}{cc} & F\left(\bar{x}+\tilde{x},\bar{y}+\tilde{y}\right)-F\left(\bar{x},\bar{y}\right)=\tilde{x}\left(\bar{y}-1\right)+\left(\bar{x}+\kappa \right)\tilde{y}+\tilde{x}\tilde{y},\\ & G\left(\bar{x}+\tilde{x},\bar{y}+\tilde{y}\right)-G\left(\bar{x},\bar{y}\right)=\left(1-\nu \right)\left[\tilde{x}\left(1-\bar{y}\right)-\bar{x}\tilde{y}-\tilde{x}\tilde{y}\right]-\left(\kappa +\mu +\nu \right)\tilde{y},\\ & \left(\bar{y}+\tilde{y}\right)\left(1-\bar{y}-\tilde{y}\right)-\bar{y}\left(1-\bar{y}\right)=\tilde{y}\left(1-2\bar{y}\right)-{\tilde{y}}^{2}. \end{array}$$

Now we insert the Taylor expansions of the outer variables around h=0. Because h=ϵτ, we have

$$\begin{array}{cc} \bar{x}\left(\epsilon \tau \right) & ={\bar{x}}_{0}\left(0\right)+\epsilon \left(\tau {\bar{x}}_{0}^{\prime }\left(0\right)+{\bar{x}}_{1}\left(0\right)\right)+O\left(\epsilon^{2}\right)=1+\epsilon \left(\tau {\bar{x}}_{0}^{\prime }\left(0\right)+{\bar{x}}_{1}\left(0\right)\right)+O\left(\epsilon^{2}\right),\\ \bar{y}\left(\epsilon \tau \right) & ={\bar{y}}_{0}\left(0\right)+\epsilon \left(\tau {\bar{y}}_{0}^{\prime }\left(0\right)+{\bar{y}}_{1}\left(0\right)\right)+O\left(\epsilon^{2}\right)=y_{0}^{*}+\epsilon \left(\tau {\bar{y}}_{0}^{\prime }\left(0\right)+{\bar{y}}_{1}\left(0\right)\right)+O\left(\epsilon^{2}\right), \end{array}$$

with $y_{0}^{*}={\bar{y}}_{0}\left(0\right)=1/B$ and ${\bar{x}}_{0}^{\prime }\left(0\right)=-\mu /B$. We also expand the corrections:

$$\tilde{x}\left(\tau ,\epsilon \right)={\tilde{x}}_{0}\left(\tau \right)+\epsilon {\tilde{x}}_{1}\left(\tau \right)+O\left(\epsilon^{2}\right),\;\tilde{y}\left(\tau ,\epsilon \right)={\tilde{y}}_{0}\left(\tau \right)+\epsilon {\tilde{y}}_{1}\left(\tau \right)+O\left(\epsilon^{2}\right).$$

Substituting the expansions into (A2a) and collecting orders O(1/ϵ), we have $d{\tilde{x}}_{0}/d\tau =0$. With ${\tilde{x}}_{0}\left(\infty \right)=0$ this forces ${\tilde{x}}_{0}\left(\tau \right)\equiv 0$. Collecting orders O(1), using ${\tilde{x}}_{0}=0$ and the leading behaviours $\bar{x}\left(\epsilon \tau \right)=1+O\left(\epsilon \right)$, $\bar{y}\left(\epsilon \tau \right)=y_{0}^{*}+O\left(\epsilon \right)$, we obtain

(A3) $$\frac{d{\tilde{x}}_{1}}{d\tau }=\left(1+\kappa \right){\tilde{y}}_{0}\left(\tau \right).$$

In (A2b), the left-hand side becomes

$$\frac{d\tilde{y}}{d\tau }=\frac{d{\tilde{y}}_{0}}{d\tau }+\epsilon \frac{d{\tilde{y}}_{1}}{d\tau }+O\left(\epsilon^{2}\right).$$

For the right-hand side, we separate orders. The O(1) term from the *G*-difference $\left[G\left(\bar{x}+\tilde{x},\bar{y}+\tilde{y}\right)-G\left(\bar{x},\bar{y}\right)\right]$ is

$$\left(1-\nu \right)\left(1-y_{0}^{*}\right){\tilde{x}}_{0}-B{\tilde{y}}_{0}=-B{\tilde{y}}_{0},$$

since ${\tilde{x}}_{0}=0$. Equating both sides at O(1) gives $\frac{d{\tilde{y}}_{0}}{d\tau }=-B{\tilde{y}}_{0}$. At O(ϵ), the contribution from $\left[G\left(\bar{x}+\tilde{x},\bar{y}+\tilde{y}\right)-G\left(\bar{x},\bar{y}\right)\right]$ is

$$\begin{array}{cc} & \left[\left(1-\nu \right)\left(1-y_{0}^{*}\right){\tilde{x}}_{1}-B{\tilde{y}}_{1}\right]-\left(1-\nu \right){\tilde{y}}_{0}\left(\tau {\bar{x}}_{0}^{\prime }\left(0\right)+{\bar{x}}_{1}\left(0\right)\right)-\left(1-\nu \right){\tilde{x}}_{1}{\tilde{y}}_{0}. \end{array}$$

The term $-\epsilon \nu [\left(\bar{y}+\tilde{y}\right)\left(1-\bar{y}-\tilde{y}\right)-\bar{y}\left(1-\bar{y}\right)]$ contributes at O(ϵ) as $\left[-\nu {\tilde{y}}_{0}\left(1-2y_{0}^{*}\right)+\nu {\tilde{y}}_{0}^{2}\right]$. Any terms containing $\tau {\bar{y}}_{0}^{\prime }\left(0\right)$ would appear multiplied by an extra ϵ and thus belong to $O(\epsilon^{2})$. Equating the O(ϵ) terms on both sides of (A2b) gives:

(A4) $$\begin{array}{cc} \frac{d{\tilde{y}}_{1}}{d\tau }= & -B{\tilde{y}}_{1}+\left(1-\nu \right)\left(1-y_{0}^{*}\right){\tilde{x}}_{1}-\left(1-\nu \right){\tilde{y}}_{0}\left(\tau \right)\left(\tau {\bar{x}}_{0}^{\prime }\left(0\right)+{\bar{x}}_{1}\left(0\right)\right)\\ \; & -\left(1-\nu \right){\tilde{x}}_{1}{\tilde{y}}_{0}-\nu {\tilde{y}}_{0}\left(1-2y_{0}^{*}\right)+\nu {\tilde{y}}_{0}^{2}. \end{array}$$

Initial conditions. The composite solution must satisfy the original initial conditions (x(0),y(0))=(1,0). Adopting the composite expansions, we have

$$\begin{array}{cc} 1 & =\left[{\bar{x}}_{0}\left(0\right)+\epsilon {\bar{x}}_{1}\left(0\right)+\cdots \right]+\left[{\tilde{x}}_{0}\left(0\right)+\epsilon {\tilde{x}}_{1}\left(0\right)+\cdots \right]\\ & =1+\epsilon \left({\bar{x}}_{1}\left(0\right)+{\tilde{x}}_{1}\left(0\right)\right)+O\left(\epsilon^{2}\right),\\ 0 & =\left[{\bar{y}}_{0}\left(0\right)+\epsilon {\bar{y}}_{1}\left(0\right)+\cdots \right]+\left[{\tilde{y}}_{0}\left(0\right)+\epsilon {\tilde{y}}_{1}\left(0\right)+\cdots \right]\\ & =\left(y_{0}^{*}+{\tilde{y}}_{0}\left(0\right)\right)+\epsilon \left({\bar{y}}_{1}\left(0\right)+{\tilde{y}}_{1}\left(0\right)\right)+O\left(\epsilon^{2}\right). \end{array}$$

Since ${\tilde{y}}_{0}\left(0\right)=-y_{0}^{*}$ satisfies the O(1) condition, the O(ϵ) conditions become

$${\bar{x}}_{1}\left(0\right)+{\tilde{x}}_{1}\left(0\right)=0,\;{\bar{y}}_{1}\left(0\right)+{\tilde{y}}_{1}\left(0\right)=0.$$

The decay conditions ${\tilde{x}}_{1}\left(\infty \right)={\tilde{y}}_{1}\left(\infty \right)=0$ select the unique physically relevant solution.

Explicit solutions. Equation (A3) together with ${\tilde{y}}_{0}\left(\tau \right)=-\frac{1}{B}\;e^{-B\tau }$ is easily integrated, giving the decaying solution

(A5) $${\tilde{x}}_{1}\left(\tau \right)=\frac{1+\kappa }{B^{2}}\;e^{-B\tau }.$$

Consequently, the initial value for the first-order outer solution ${\bar{x}}_{1}\left(h\right)$ is obtained

(A6) $${\bar{x}}_{1}\left(0\right)=-{\tilde{x}}_{1}\left(0\right)=-\frac{1+\kappa }{B^{2}}.$$

On the other hand, the equation for ${\tilde{y}}_{1}\left(\tau \right)$ in (A4) becomes, after substituting the explicit forms of ${\tilde{y}}_{0}\left(\tau \right),\;{\tilde{x}}_{1}\left(\tau \right)$ and the constants,

(A7) $$\frac{d{\tilde{y}}_{1}}{d\tau }+B\;{\tilde{y}}_{1}=-\frac{(1-\nu )\mu }{B^{2}}\;\tau e^{-B\tau }+\frac{\left(B-2)(1+\kappa +\nu \mu \right)}{B^{3}}\;e^{-B\tau }+\frac{1+\kappa +\nu \mu }{B^{3}}\;e^{-2B\tau }.$$

All terms on the right-hand side are of the form $\tau e^{-B\tau }$, $e^{-B\tau }$ or $e^{-2B\tau }$. Equation (A7) can be solved by the integrating factor $e^{B\tau }$:

$$\frac{d}{d\tau }\left({\tilde{y}}_{1}e^{B\tau }\right)=-\frac{(1-\nu )\mu }{B^{2}}\;\tau +\frac{\left(B-2)(1+\kappa +\nu \mu \right)}{B^{3}}+\frac{1+\kappa +\nu \mu }{B^{3}}\;e^{-B\tau }.$$

Integrating and imposing the initial value ${\tilde{y}}_{1}\left(0\right)=-{\bar{y}}_{1}\left(0\right)$, we deduce the resulting first-order corrected inner solution

(A8) $${\tilde{y}}_{1}\left(\tau \right)=\left[-\frac{(1-\nu )\mu }{2B^{2}}\;\tau^{2}+\frac{\left(B-2)(1+\kappa +\nu \mu \right)}{B^{3}}\;\tau -\frac{1+\kappa +\nu \mu }{B^{4}}\;e^{-B\tau }+K\right]e^{-B\tau },$$

with $K=\frac{1}{B^{2}}-\frac{\mu (1-\nu )(2B-1)}{B^{4}}$.

### Appendix A.2. Positivity of Outer Solution

Although the leading-order composite solution provides a good approximation of the original MM reaction, it may result in a negative outer concentration of the product [P]. To illustrate, we simply choose the initial time h=0. Based on the law of mass conservation, the outer part of the product concentration at h=0 is negative, ${\left[P\right]|}_{h=0}=S_{0}\left[1-{\bar{x}}_{0}\left(0\right)\right]-E_{0}{\bar{y}}_{0}\left(0\right)=-E_{0}{\bar{y}}_{0}\left(0\right)<0$, by recalling that ${\bar{x}}_{0}\left(0\right)=1$ and ${\bar{y}}_{0}\left(0\right)=1/B>0$.

To preserve the positivity of the outer concentrations, an independent asymptotic expansion for the product concentration [P] is introduced. Define a dimensionless product concentration $z\left(h\right)=\left[P\right]/S_{0}$, whose evolution is governed by

(A9) $$\frac{dz}{dh}=\mu y-\nu z\left(1-y\right),\;z\left(0\right)=0.$$

Although this equation does not contain the small parameter ϵ explicitly, it inherits a two-scale structure by coupling to the fast variable $y=y^{\epsilon }\left(h\right)$.

Analogously, we seek a matched asymptotic expansion of z(h) in the form $z^{\epsilon }\left(h\right)={\bar{z}}_{0}\left(h\right)+\epsilon {\bar{z}}_{1}\left(h\right)+\cdots +{\tilde{z}}_{0}\left(\tau \right)+\epsilon {\tilde{z}}_{1}\left(\tau \right)+\cdots$. The leading-order outer solution ${\bar{z}}_{0}\left(h\right)$ satisfies

(A10) $$\frac{d{\bar{z}}_{0}}{dh}=\mu {\bar{y}}_{0}-\nu {\bar{z}}_{0}\left(1-{\bar{y}}_{0}\right),\;{\bar{z}}_{0}\left(0\right)=0.$$

Since the product formation is negligible on the fast time scale, the inner correction at the leading order vanishes, ${\tilde{z}}_{0}\left(\tau \right)\equiv 0$. The first-order outer solution ${\bar{z}}_{1}\left(h\right)$ obeys

(A11) $$\frac{d{\bar{z}}_{1}}{dh}=\mu {\bar{y}}_{1}-\nu {\bar{z}}_{1}\left(1-{\bar{y}}_{0}\right)+\nu {\bar{z}}_{0}{\bar{y}}_{1},$$

with the initial condition ${\bar{z}}_{1}\left(0\right)=0$ obtained from matching with the inner layer. The first-order corrected inner solution is given explicitly by

(A12) $${\tilde{z}}_{1}\left(\tau \right)=\frac{\mu }{B^{2}}\left(e^{-B\tau }-1\right).$$

The composite solution z(h) becomes

(A13) $$z^{\epsilon }\left(h\right)={\bar{z}}_{0}\left(h\right)+\epsilon {\bar{z}}_{1}\left(h\right)+\epsilon {\tilde{z}}_{1}\left(\tau \right)+O\left(\epsilon^{2}\right).$$

This independent treatment of the product avoids the negative values that would arise from simply substituting the outer solutions for x(h) and y(h) into the law of mass conservation. With this treatment, the outer part of product concentration $\left[P\right]\left(h\right)=S_{0}\bar{z}\left(h\right)$ remains strictly non-negative for all h≥0, as shown in Figure 1d and Figure A1d.

## Appendix B. Steady States of MM Reaction

In this part, we derive the steady states of the outer solution and the full MM kinetics in Section 3 respectively. Firstly, the steady state ${\left({\bar{x}}_{e},{\bar{y}}_{e}\right)}^{T}$ of the outer solution to the reduced MM kinetics (31a) and (31b) satisfies

$$\begin{array}{cc} \; & -{\bar{x}}_{e}\left(1-{\bar{y}}_{0}\right)+\kappa {\bar{y}}_{e}=0,\\ \; & \left(1-\nu \right){\bar{x}}_{e}\left(1-{\bar{y}}_{e}\right)+\nu -\left(\kappa +\mu +\nu \right){\bar{y}}_{e}=0. \end{array}$$

Substituting the relation ${\bar{x}}_{e}=\frac{\kappa {\bar{y}}_{e}}{1-{\bar{y}}_{e}}$ deduced from the first equation into the second, we have:

$${\bar{x}}_{e}=\frac{\kappa \nu }{\kappa \nu +\mu },\;{\bar{y}}_{e}=\frac{\nu }{\kappa \nu +\mu +\nu }.$$

On the other hand, regarding the dimensionless Equation (30) of the full MM reaction, the steady state ${\left(x_{e}^{\epsilon },y_{e}^{\epsilon }\right)}^{T}$ satisfies

$$\begin{array}{cc} \; & -x_{e}^{\epsilon }\left(1-y_{e}^{\epsilon }\right)+\kappa y_{e}^{\epsilon }=0,\\ \; & \left(1-\nu \right)x_{e}^{\epsilon }\left(1-y_{e}^{\epsilon }\right)+\nu -\left(\kappa +\mu +\nu \right)y_{e}^{\epsilon }-\epsilon \nu y_{e}^{\epsilon }\left(1-y_{e}^{\epsilon }\right)=0. \end{array}$$

By inserting the first equation and simplifying, the second one becomes

$$\left(1-\nu \right)\kappa y_{e}^{\epsilon }+\nu -\left(\kappa +\mu +\nu \right)y_{e}^{\epsilon }-\epsilon \nu y_{e}^{\epsilon }\left(1-y_{e}^{\epsilon }\right)=0,$$

or equivalently,

(A14) $$-\left(\kappa \nu +\mu +\nu \right)y_{e}^{\epsilon }+\nu =\epsilon \nu y_{e}^{\epsilon }\left(1-y_{e}^{\epsilon }\right).$$

Recall that ${\bar{y}}_{e}=\nu /\left(\kappa \nu +\mu +\nu \right)$. Writing $y_{e}^{\epsilon }={\bar{y}}_{e}+\delta$, and substituting it into Equation (A14), we have:

$$-\left(\kappa \nu +\mu +\nu \right)\left({\bar{y}}_{e}+\delta \right)+\nu =-\left(\kappa \nu +\mu +\nu \right)\delta =\epsilon \nu \left({\bar{y}}_{e}+\delta \right)\left(1-{\bar{y}}_{e}-\delta \right).$$

The left-hand side simplifies because $-\left(\kappa \nu +\mu +\nu \right){\bar{y}}_{e}+\nu =0$. Expand the right-hand side $\epsilon \nu \left[{\bar{y}}_{e}\left(1-{\bar{y}}_{e}\right)+\delta \left(1-2{\bar{y}}_{e}\right)-\delta^{2}\right]$. Since δ is expected to be O(ϵ), we neglect $\delta^{2}$ at the leading order:

$$-\left(\kappa \nu +\mu +\nu \right)\delta =\epsilon \nu {\bar{y}}_{e}\left(1-{\bar{y}}_{e}\right)+O\left(\epsilon^{2}\right).$$

Then, $\delta =-\frac{\epsilon \nu {\bar{y}}_{e}\left(1-{\bar{y}}_{e}\right)}{\kappa \nu +\mu +\nu }+O\left(\epsilon^{2}\right)$. Therefore, the asymptotic expansion of the steady state to the full MM kinetics is finally obtained as

$$y_{e}^{\epsilon }={\bar{y}}_{e}-\frac{\epsilon \nu {\bar{y}}_{e}\left(1-{\bar{y}}_{e}\right)}{\kappa \nu +\mu +\nu }+O\left(\epsilon^{2}\right),\;x_{e}^{\epsilon }=\frac{\kappa y_{e}^{\epsilon }}{1-y_{e}^{\epsilon }}.$$

## Appendix C. Numerical Results of First-Order Asymptotic Expansions

Here we provide additional numerical results for the first-order asymptotic expansions of the MM reaction.

Compared to the leading-order results in Figure 1, the first-order composite solutions presented in Figure A1 show a significant improvement in accuracy. For all kinetic variables in Figure A1a–d, the first-order composite curves are visually indistinguishable from the exact solutions, and a similar near-perfect overlap is observed for the four thermodynamic quantities in Figure A1e–h.

The error analysis in Figure A2 quantifies this improvement and, more importantly, reveals a marked distinction in the convergence behavior of different thermodynamic quantities. As shown in Figure A2a,b, the errors for x(h) and y(h) are now closely aligned with the reference line $O(\epsilon^{2})$, confirming that the first-order expansion successfully improves the accuracy of the kinetic variables to second order. The enthalpy H(h), owing to its linear dependence on the concentrations, also achieves $O(\epsilon^{2})$ convergence. In contrast, the entropy Ent(h) and relative entropy F(h) in Figure A2c,d remain bounded by the O(ϵ) reference line and do not show an improvement in the convergence order. This is a direct consequence of the logarithmic singularities in their gradients, which amplify the already small concentration errors within the initial layer. Remarkably, the entropy production rate epr(h) escapes this limitation. Its MAE and $L_{\infty }$ errors follow the $O(\epsilon^{2})$ line. This unique behavior originates from the specific algebraic structure of epr, where the factor $\left(R^{+}-R^{-}\right)$ in front of the logarithm provides an additional order of smallness that cancels the gradient divergence, preserving the accuracy of the first-order concentration expansion.
